# GSK-3β controls NF-kappaB activity via IKKγ/NEMO

**DOI:** 10.1038/srep38553

**Published:** 2016-12-08

**Authors:** Senad Medunjanin, Lisa Schleithoff, Christian Fiegehenn, Soenke Weinert, Werner Zuschratter, Ruediger C. Braun-Dullaeus

**Affiliations:** 1 Internal Medicine/Cardiology and Angiology, Magdeburg University, Magdeburg, Germany; 2 Leibniz Institute for Neurobiology, Magdeburg, Germany

## Abstract

The NF-κB signaling pathway is central for the innate immune response and its deregulation is found in multiple disorders such as autoimmune, chronic inflammatory and metabolic diseases. IKKγ/NEMO is essential for NF-κB activation and NEMO dysfunction in humans has been linked to so-called progeria syndromes, which are characterized by advanced ageing due to age-dependent inflammatory diseases. It has been suggested that glycogen synthase kinase-3β (GSK-3β) participates in NF-κB regulation but the exact mechanism remained incompletely understood. In this study, we identified NEMO as a GSK-3β substrate that is phosphorylated at serine 8, 17, 31 and 43 located within its N-terminal domain. The kinase forms a complex with wild-type NEMO while point mutations of NEMO at the specific serines abrogated GSK-3β binding and subsequent phosphorylation of NEMO resulting in its destabilization. However, K63-linked polyubiquitination was augmented in mutated NEMO explaining an increased binding to IKKα and IKKβ. Even IκBα was found degraded. Still, TNFα-stimulated NF-κB activation was impaired pointing towards an un-controlled signalling process. Our data suggest that GSK-3β is critically important for ordered NF-κB signalling through modulation of NEMO phosphorylation.

NF-κB represents a family of evolutionarily conserved transcription factors consisting of five members: c-Rel, RelA (p65), RelB, p50 (NF-κB1/p105 precursor), and p52 (NF-κB2/p100 precursor)^1^. In resting cells NF-κB is rendered inactive within the cytoplasm through association with inhibitory IκB proteins. Various inflammatory stimuli can trigger the activation of the IκB kinase (IKK) complex, which consists of the regulatory subunit NF-κB Essential Modifier (NEMO/IKKγ) and two catalytic subunits (IKKα and IKKβ)^2^. Upon IKK activation, IκB is phosphorylated and, subsequently, targeted for rapid proteasomal degradation, thus liberating NF-κB for nuclear translocation, enhanced DNA binding and transcriptional regulation^3^. NEMO is an adaptor protein involved in activation of the IKK kinases and has been shown to be critically important for the canonical^4^ and stress-induced NF-κB pathway^5^. Ablation of NEMO in mice resulted in a lack of detectable NF-κB DNA-binding activity and a lethal embryonic phenotype because of severe liver damage due to massive apoptosis^6^.

Glycogen synthase kinase-3 (GSK-3) is a serine/threonine kinase that exists as two highly similar mammalian isoforms (GSK-3α and GSK-3β)^7^. Although highly homologous within their kinase domains, these isoforms are functionally not identical. Ablation of the GSK-3β isoform in mice resulted in a lethal embryonic phenotype^8^, indicating the inability of GSK-3α to rescue the GSK-3β-null mice. GSK-3β is constitutively active but several mechanisms contribute to controlling its actions. Inhibitory phosphorylation by Akt and other kinases occurs at serine 9. GSK-3β is also regulated by protein complex formation, well-known in the cytosolic Wnt signalling pathway, where the kinase associates with a large protein complex and phosphorylates β-catenin to promote its degradation^7^.

Similar to NEMO-deficient animals, mice lacking GSK-3β die during development due to multifocal haemorrhagic degeneration of the liver^8^. These data indicate that loss of GSK-3β results in defective NF-κB signalling in response to TNFα stimulation. GSK-3β’s role in constitutive NF-κB reporter activity and target gene expression has further been demonstrated in pancreatic cancer models^9^. Furthermore, GSK-3β’s requirement for TNF-α-induced transcription and promoter recruitment of p65 to a subset of NF-κB-regulated genes could be demonstrated^10^. However, during our investigations on the signalling interaction between GSK-3β and NEMO we were surprised to find that mutations of GSK-3β phosphorylation sites in NEMO leads to a strong reduction of NEMO protein level and, consequently, reduction of NF-κB activation. We, therefore, hypothesized that NEMO represents a critical link between GSK-3β and NF-κB transcriptional activity.

## Results

### GSK-3β is involved in NF-κB activation

**Figure 1:**
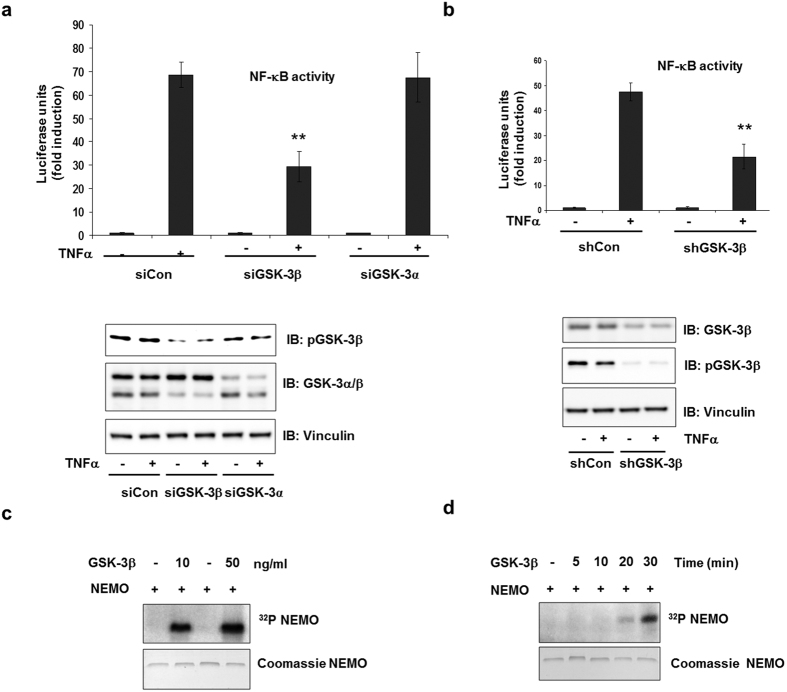
GSK-3β is involved in NF-κB activation. (**a**) HEK293 cells were transfected either with control siRNA (siCon) or with siRNA-targeting GSK-3β (siGSK-3β) or siRNA-targeting GSK-3α (siGSK-3α). After 24 h stimulation with TNFα (1ng/ml), NF-κB-dependent gene expression was quantified by measurement of the luciferase activity. Fold induction is the ratio of stimulated to unstimulated cells (**P < 0.01). Cell lysates were immunoblotted with the antibodies indicated. (**b**) HEK293 cells were stably transfected either with control shRNA (shCtr) or with shRNA-targeting GSK-3β (shGSK-3β). After 24 h stimulation with TNFα (1ng/ml), NF-κB-dependent gene expression was quantified by measurement of the luciferase activity. Cell lysates were immunoblotted with the antibodies indicated. (**c**) *In vitro* kinase assay using recombinant human 1 μg NEMO as substrate for GSK-3β (0.01 μg or 0.05 μg). (**d**) GSK-3β (0.01 μg) was incubated with 1 μg recombinant human NEMO and 10 μCi of [γ-32P]ATP at 30 °C for different time periods.

To clarify the role of GSK-3 in NF-κB function, we used several small interfering RNA (siRNA) to knockdown GSK-3 levels. In the presence of the specific siRNA, GSK-3 protein levels were reduced (Fig. 1a). HEK293 cells stably transfected with a luciferase reporter gene under the control of a NF-κB response element were treated with TNFα with or without the GSK-3 siRNA. TNFα treatment resulted in a ~70-fold induction of luciferase activity, which was significantly hampered by transfection of siGSK-3β, but not by siGSK-3α (Fig. 1a and Supplementary Figure 1a). The inhibition of TNFα-dependent NF-κB transcriptional activation by siGSK-3β suggested a role of active GSK-3β in NF-κB activation. Similar results were obtained using the GSK-3 inhibitor SB 216763 (data not shown) or shGSK-3β (Fig. 1b). Since phosphorylation is an important step in NEMO regulation^11^,^12^, we investigated whether NEMO is a target of GSK-3β. Using recombinant human NEMO (rhNEMO) and GSK-3β, we were able to visualize NEMO phosphorylation by GSK-3β in a dose- and time-dependent manner by means of an *in vitro* kinase assay (Fig. 1c,d).

### GSK-3β associates with human NEMO

**Figure 2:**
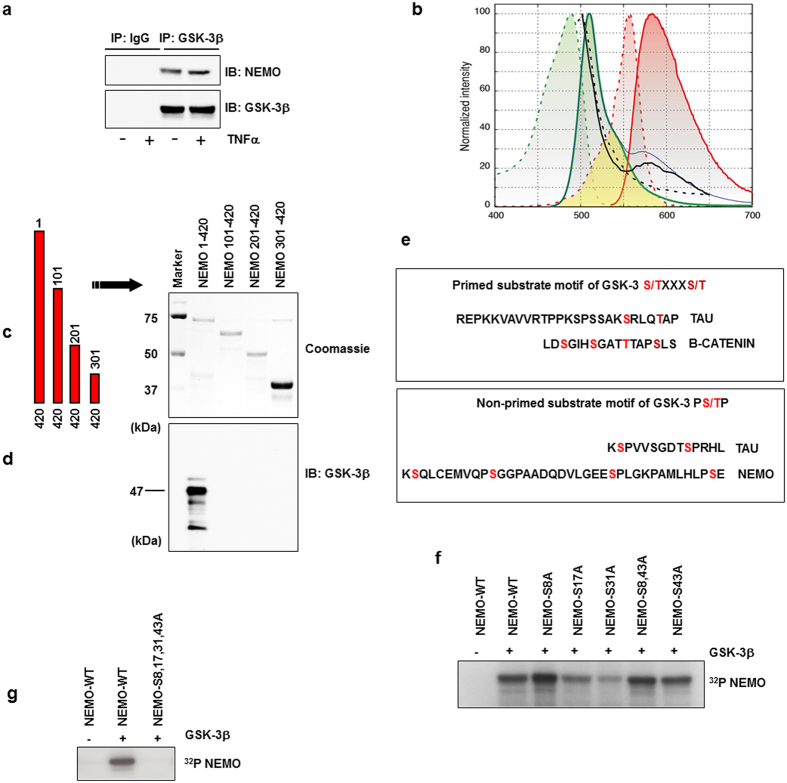
GSK-3β associates with human NEMO. (**a**) CoIP of NEMO and GSK-3β from lysates of HEK293 cells after stimulation with TNFα (10 ng/ml) for 30 min. (**b**) Lambda scans of co-transfected NEMO (TagRFP) and GSK-3β (GFP) in HEK293 showed facilitated emission of NEMO-TagRFP (black curve) after excitation of GFP-GSK-3β with blue light of 476 nm by confocal laser scanning microscopy. Excitation (dotted lines) and emission (bold lines) spectra of GFP (green curves) and TagRFP (red) with the spectral overlap (yellow area) between GFP emission and TagRFP excitation showed that, theoretically, an increase of approximately 30% of TagRFP emission (blue curve) could be expected. We found an increase of approximately 20% (black curve) in our FRET experiments that could be abolished after acceptor bleaching with strong 561 nm laser light (black dotted curve). (**c**) Schematic representation of NEMO constructs used. (**d**) CoIP from lysates of HEK293 cells transfected with the constructs as indicated. Wild-type NEMO (NEMO 1-420) or NEMO deletion mutants after immunoprecipitation with GST beads (Coomassie staining). (**e**) Motif of primed known GSK-3 phosphorylation sites (upper box). Primed serine residues are labeled in *dark red*; GSK-3 phosphorylation sites are labeled in light red. Motifs for non-primed GSK-3 (lower panel) substrates are labeled in *light red*. NEMO serine residues labeled in *light red* may represent direct targets for GSK-3. (**f**) *In vitro* kinase assay using wild-type and mutant GST-NEMO fusion proteins (1 μg) as substrates for GSK-3β (0.01 μg). (**g**) *In vitro* kinase assay using wild-type and mutant GST-NEMO fusion proteins (1 μg) as substrates for GSK-3β (0.01 μg).

GSK-3β has been reported as a docking kinase^7^ able to form complexes with substrates in different signalling pathways. To further clarify if NEMO is a substrate of GSK-3β, we investigated whether NEMO and GSK-3 physically associate *in vitro*. The interaction of GSK-3β and NEMO was assessed by immune-precipitation of endogenous GSK-3β and the subsequent analysis of the immune complexes for the presence of NEMO or GSK-3β. We observed an association of GSK-3β and NEMO in untreated cells that was not modulated by stimulation with TNFα (Fig. 2a). The results of the immune-precipitation were confirmed by FRET analysis (Fig. 2b), and FLIM-FRET analysis (Supplementary Figure 1b). Furthermore, co-localisation of endogenous NEMO with endogenous GSK-3β was observed by STED microscopy (Supplementary Figure 1c). In addition to HEK293 cells, interaction of NEMO and GSK-3 could be confirmed in MCF-7, NIH3T3 mouse cells, and human macrophages (Supplementary Figure 1 d–f). We then determined the domain of NEMO interacting with GSK-3β. A GST pull-down assay was performed. GST-fused NEMO and different deletion mutants of NEMO (Fig. 2c) were mixed with cell lysates containing GSK-3β. After overnight incubation, GST-NEMO was eluted from the resin and GSK-3β within the elutant was analyzed by immune blotting. A GSK-3β signal was detected only when full-length NEMO was present (Fig. 2d) indicating that the N-terminal domain of NEMO is essential for the interaction with GSK-3β *in vitro*. Amino acid sequence comparison of the N-terminal domain with known GSK-3β substrates^13^,^14^ revealed several putative GSK-3 phosphorylation sites within the NEMO N-terminal domain (Fig. 2e). In order to identify the sites phosphorylated by GSK-3β, we generated series of phosphorylation site-specific NEMO mutants in which the serine residues 8, 17, 31, and 43 were mutated to alanine individually (S8A, S17A, S31A, S43A, respectively). Furthermore, we generated a mutant in which both flanked serines 8 and 43 were replaced by alanine (S8A, S43A, respectively), because GSK-3 preferentially phosphorylates substrates on serine and threonine residues followed by proline in a relay fashion^14^,^15^. We tested GSK-3β for its ability to phosphorylate these NEMO fusion proteins by means of *in vitro* kinase assays. Surprisingly, phosphorylation of the S8A mutant seemed augmented in comparison to wild-type NEMO (Fig. 2f). Phosphorylation of the S17A and S31A mutants was markedly reduced compared to wild-type NEMO, whereas phosphorylation of either S43A or S8, 43A was similar to the wild-type protein (Fig. 2f). *In vitro* phosphorylation of a mutant in which all four serines (8, 17, 31, 43) had been exchanged by alanine revealed no phosphorylation by GSK-3β at all (Fig. 2g).

### GSK-3β-mediated NEMO phosphorylation is required for NF-κB activity

**Figure 3:**
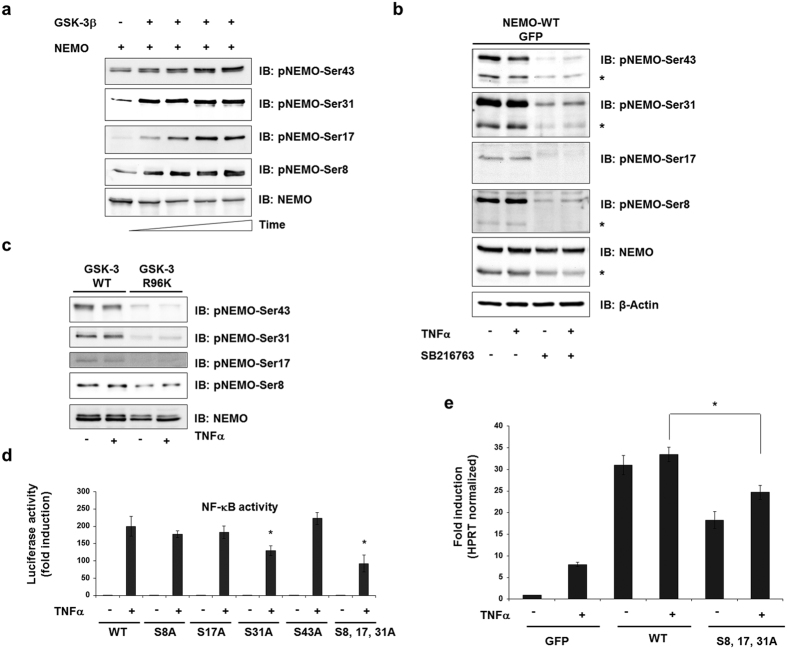
GSK-3β-mediated NEMO phosphorylation is required for NF-κB activity. (**a**) *In vitro* kinase assay using recombinant human NEMO (1 μg) as substrate for GSK-3 (0.01 μg) in a time-dependent manner. The same amounts of proteins are immunoblotted with the antibodies indicated. (**b**) HEK293 cells were transfected wild-type NEMO. After treatment with the GSK-3 inhibitor SB216763 (1 μM) for 12 h, cells were stimulated with TNFα (10 ng/ml) for 1 h. Cell lysates were immunoblotted with the antibodies indicated. Asteriscs indicate phospho-endogenous NEMO. (**c**) HEK293 cells were transfected with wild-type GSK-3β or mutant GSK-3β-R96K and cell lysates were assayed for the expression of the phosphorylated form of NEMO. (**d**) HEK293 cells were transfected with expression vectors carrying wild-type and mutant NEMO (1 μg each), NF-κB-dependent gene expression was quantified by measuring luciferase activity. -Fold induction is the ratio of stimulated to unstimulated cells (*P < 0.05). (**e**) HEK293 cells were transfected either with wild-type NEMO or with mutated NEMO. The cells were treated—or not—with TNFα (10 ng/ml) for 24 h, and analyzed for the expression of NF-κB-target gene, IL8 by quantitative RT–PCR. (*P < 0.05 vs. WT).

Phosphorylation of NEMO was further investigated by *in vitro* phosphorylation of recombinant NEMO using phospho-specific antibodies. For analysis of phosphorylation of NEMO on Ser8 and Ser17 by GSK-3, we generated phospho-specific antibodies (anti-phospho-NEMO-Ser8 and anti-phospho-NEMO-Ser17) while anti-phospho-NEMO-Ser31 and anti-phospho-NEMO-Ser43 were commercially available. Indeed, GSK-3β was able to phosphorylate all four serines of rhNEMO in a time-dependent manner in an *in vitro* kinase assay (Fig. 3a). To prove involvement of previously reported IKKβ^16^ in the phosphorylation of NEMO, we performed *in vitro* phosphorylation of NEMO by IKKβ. However, using phosphospecific antibodies we observed no significant increase in NEMO phosphorylation by IKKβ (Supplementary Figure 2d). The phosphorylation state of these serine residues was further studied in lysates of HEK293 cells by overexpression of wild-type NEMO. Commensurate to a constitutive active GSK-3, all serines were found phosphorylated in unstimulated cells (Fig. 3b). This phosphorylation was reduced by treatment of cells with a GSK-3 inhibitor (Fig. 3b) or transfection of a kinase-inactive form of GSK-3β (Fig. 3c). We then studied the impact of the four putative GSK-3 phosphorylation sites on NF-κB transcriptional activity. For this purpose the NEMO mutants were overexpressed in HEK293 cells stably transfected with a 5xNF-κB response element and NF-κB activity was determined. While overexpression of the NEMO mutant S31A resulted in a considerable reduction of TNFα-induced luciferase activity, overexpression of NEMO S43A revealed no difference but rather an increase of NF-κB activity after TNFα stimulation in comparison to wild-type NEMO (Fig. 3d). Consequently, a triple mutant S8, 17, 31A was generated and overexpressed, which resulted in the strongest reduction of NF-κB activity indicating that these serine residues, determined as GSK-3β phosphorylation sites, were required for full transcriptional activation of NF-κB (Fig. 3d). This triple mutant was used for further investigation of the role of GSK-3β in NEMO regulation. Recent reports have described that inhibition of GSK-3β leads to a reduced expression of NF-κB target genes^17^. Therefore, we tested the mRNA expression of pro-inflammatory NF-κB target genes IL8^18^,^19^ and interleukin-1beta^20^ both expressed in HEK293 cells after transfection of the NEMO triple mutant. Indeed, reduced expression of mRNA in cells transfected with mutated NEMO in comparison to wild-type NEMO was determined (Fig. 3e and Supplementary Figure 2e).

### GSK-3β modulates NEMO protein stability

**Figure 4:**
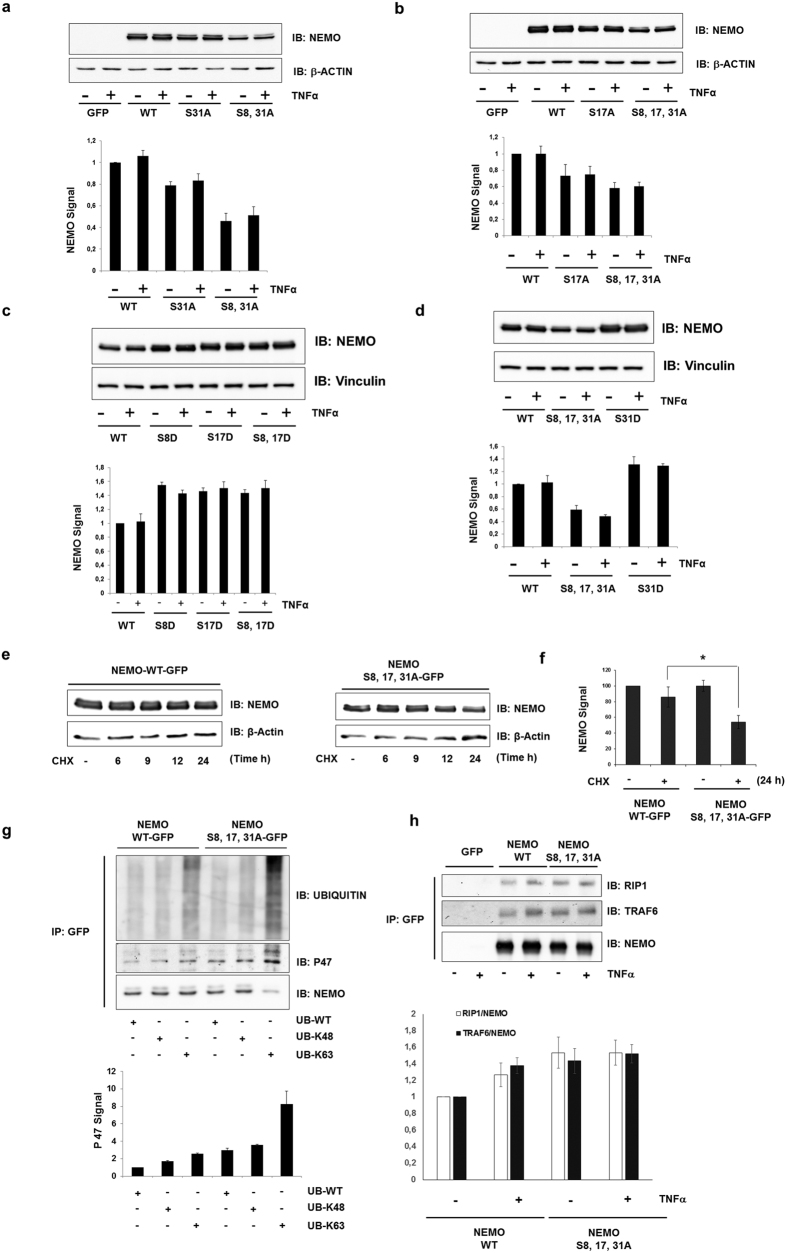
GSK-3β-mediated phosphorylation induces NEMO stabilization. (**a**) The lysates from cells transfected with NEMO constructs were immunoblotted (IB) with the antibodies indicated. β-Actin was used as a loading control. Quantitative analysis (lower panel) of NEMO. (Normalized to β-actin, the ratio at control cells was set as 1.) (**b**) Cells were lysed 48 h after transfection and immunoblotted (IB) with anti-NEMO. β-Actin was used as a loading control. Quantitative analysis (lower panel) of NEMO. (Normalized to β-actin, the ratio at control cells was set as 1.) (**c,d**) The lysates from cells transfected with NEMO constructs were immunoblotted (IB) with the antibodies indicated. Quantitative analysis (lower panel) of NEMO. (Normalized to Vinculin, the ratio at control cells was set as 1.). Vinculin was used as a loading control. (**e, f**) NEMO protein turnover was evaluated by cycloheximide half-life experiments. Immunoblot analysis of whole-cell lysates of HEK293 cells transfected with vectors encoding GFP-tagged wild-type or mutant NEMO and treated with cycloheximide (CHX) (50 μM) for 0–24 h. (**g**) HEK293 lysates of cells transfected with wild-type NEMO or with the S8A, S17A, S31A mutant of NEMO in combination with various HA-tagged ubiquitin species were subjected to anti-GFP immune-precipitation followed by anti-ubiquitin, anti-P47 and anti-NEMO immunoblotting (IB). Quantitative analysis (lower panel) of P47. (Normalized to NEMO, the ratio at control cells was set as 1.) (**h**) HEK293 cells were transfected with wild-type NEMO or with the S8, 17, 31A mutant of NEMO. After 48 h of incubation, lysates from TNFα-stimulated (10 ng/ml) cells were immunoprecipitated (IP) with anti-GFP, followed by immunoblotting (IB) with anti-TRAF6 and anti-RIP1. Quantitative analysis (lower panel) of RIP1 and TRAF6. (Normalized to immunoprecipitated NEMO, the ratio at control cells was set as 1).

We constructed several GFP-tagged NEMO/IKKγ constructs and visualized their presentation in living cells to exclude the fact that only misfolding or intracellular misdistribution of the mutants accounted for the attenuation of TNFα-induced NF-κB activation. We analyzed the protein expression of the different NEMO mutants and observed a considerable decrease of all NEMO mutants in comparison to wild-type NEMO (Fig. 4a,b). Similarly, reduced NEMO expression was observed using siGSK-3β which could be rescued by co-transfection of wild-type GSK-3β but not GSK-3β kinase inactive mutant (R96K) (Supplementary Figure 2b). In addition, we generated a set of mutants where serines were mutated to aspartic acid which resulted in an increase of NEMO expression indicating that these serine residues, determined as GSK-3β phosphorylation sites, were required for NEMO stability (Fig. 4c,d).

Altered transcriptional levels of mutated NEMO or wild-type NEMO after GSK-3β inhibition was first excluded by real-time PCR (not shown). Then, protein half-live was determined in HEK293 cells transfected with wild-type NEMO vs. triple mutant NEMO treated with cycloheximide and subsequent densitometric quantification of the immune-blots. While wild-type protein expression had decreased by **~**20% after 24 hours of cycloheximide treatment, the triple mutant protein was reduced to **~**50% (Fig. 4e,f) pointing towards its post-translational processing. However, Lys48-linked ubiquitination and subsequent proteasomal degradation could be excluded since treatment of HEK293 cells overexpressing wild-type NEMO or triple mutant NEMO with the proteasomal inhibitor MG132 did not result in an accumulation of K48 ubiquitinated forms of NEMO (Supplementary Figure 2c and Fig. 4g).

It is well known that K63-linked, proteasomal-independent polyubiquitination takes place in eukaryotic cells^21^,^22^ which, in the case of NEMO, is critical for modulation of NF-κB activity^23^,^24^. Therefore, we performed co-transfection of NEMO and mutant NEMO with different ubiquitin plasmids. The K63 ubiquitin mutant contained arginine substitutions at all of its lysine residues except position 63. We also included wild-type ubiquitin and a K48 ubiquitin mutant as controls. Ubiquitin immunoblotting of the immune-precipitates revealed an increased steady-state level K63-linked polyubiquitination of the NEMO mutant in comparison to the wild-type NEMO (Fig. 4g). K63-linked polyubiquitination of mutated NEMO was also supported by its increased interaction with TRAF6 and RIP1 (Fig. 4h)^25^,^26^. A recent report identified p47 as a NEMO binding protein when NEMO was conjugated to Lys63-linked polyubiquitin chains thereby triggering its lysosomal degradation^27^. Indeed, in our study co-transfection of Lys-63 ubiquitin with mutant NEMO resulted in increased polyubiquitination and interaction of the triple mutant with p47 (despite a reduced mutant NEMO protein level) (Fig. 4g) implicating that mutated NEMO undergoes lysosomal degradation.

### Interaction of mutated NEMO with GSK-3β and other components of the NF-κB pathway

**Figure 5:**
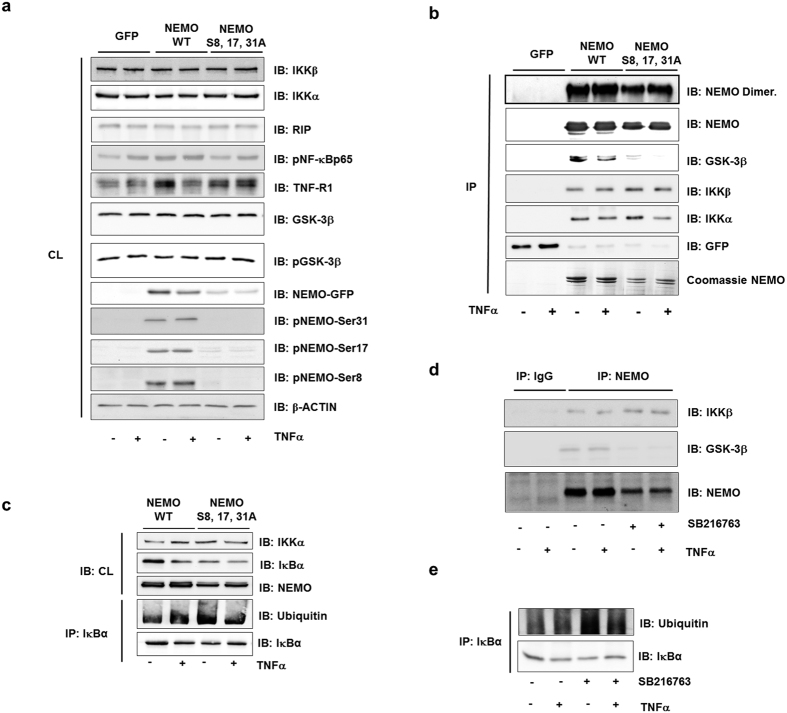
GSK-3β/IKKγ interaction modulates the NF-κB pathway. (**a**) HEK293 cells were transfected with wild-type NEMO or with the S8, 17, 31A mutant of NEMO. The lysates (CL) were assayed for protein expression before immunoprecipitation as indicated. (**b**) Lysates from TNFα-stimulated (10 ng/ml) cells were immunoprecipitated (IP) with anti-GFP, followed by immunoblotting (IB) with anti-NEMO, anti-IKKα, anti-IKKβ and anti-GSK-3β. Part of the immunoprecipitation was examined by native gel electrophoresis (upper band) to detect the dimerization of NEMO. (**c**) HEK293 cells were transfected with wild-type NEMO or with the S8, 17, 31A mutant of NEMO. The lysates (CL) were assayed for protein expression as indicated. The lysates were immunoprecipitated (IP) with anti-IKBα followed by immunoblotting (IB) with anti-ubiquitin. (**d**) After 2 h pretreatment with SB216763 (1 μM), cells were stimulated with TNFα (10 ng/ml) for 1 h. Lysates were immunoprecipitated (IP) with anti-NEMO and were followed by immunoblotting (IB) with anti-NEMO, anti-IKKβ, and anti-GSK-3β. (**e**) After 2 h pretreatment with SB216763 (1 μM), cells were stimulated with TNFα (10 ng/ml) for 1 h. Lysates were immunoprecipitated (IP) with anti-IĸBα and were followed by immunoblotting (IB) with anti-Ubiquitin and IĸBα.

NEMO interaction was examined in HEK293 cells after overexpression of its wild-type or triple-mutated form. First, we tested expression of phosphorylated NEMO and components of NF-κB in cell lysates prior to immunoprecipitation (Fig. 5a). Furthermore, phospho-specific antibodies were tested by overexpression of appropriate mutants of NEMO where serine was changed to alanine (Fig. 5a). Co-immunoprecipitation of full-length wild-type NEMO showed a clear interaction of NEMO with GSK-3β while the binding of the triple-mutated NEMO-S8, 17, 31A to GSK-3β was markedly reduced (Fig. 5b). Furthermore, we examined whether NEMO mutants are defective in homodimer formation using native PAGE gel electrophoresis. As expected, reduced expression of NEMO mutant in comparison to wild-type NEMO protein was observed (Fig. 5b upper band). Still, NEMO was able to form homodimers excluding the possibility that impeded NEMO dimerization was the reason for the reduced NF-κB activity.

Though, interaction of mutated NEMO with IKKs in resting cells and after stimulation with TNFα was still observed and even appeared increased especially in view of reduced protein levels of the mutant form (Fig. 5b). Subsequently, we were able to demonstrate a marked reduction of IκBα protein level in resting cells already, implying that IκBα is constantly degraded, as well (Fig. 5c). This degradation was a consequence of ubiquitination, as shown by an ubiquitination assay after immune-precipitation of IκBα (Fig. 5c). In accordance with these findings, pharmacologic inhibition of GSK-3β with SB216763 resulted in a discrete increase of NEMO binding to IKKβ, despite the fact that GSK-3β binding to the complex was strongly reduced (Fig. 5d). In addition, inhibition of GSK-3β led to increased ubiquitination and downregulation of IκBα (Fig. 5e). Similar results were observed using a GSK-3β kinase inactive mutant (not shown). Taken together, these results imply that constitutive GSK-3β activity in quiescent cells is necessary for ordered NF-κB activation.

## Discussion

IKKγ/NEMO plays an essential role in the activation of the canonical IKK complex. Therefore, it is not surprising that its function is tightly regulated. There is emerging evidence that NEMO is controlled by post-translational modifications as well as by its interaction with other proteins^28^.

In this study, we identified NEMO as a GSK-3β substrate that is phosphorylated at several serine residues located within the N-terminal domain. Interaction analysis of truncation mutants identified the N-terminal region of NEMO which encompasses an α-helical domain necessary for association with GSK-3β^29^. These serine residues, typically located in a GSK-3β-specific proline-rich domain, are involved in the control of NF-κB. Using NEMO mutants in which serines were replaced by alanine and which could not be phosphorylated by GSK-3β, we observed a reduction of NEMO protein expression. As a consequence, TNFα-induced NF-κB activation was hampered. Still, mutated NEMO was able to bind to IKKα and IKKβ. Even IκBα was found degraded, already in unstimulated cells, pointing towards an increased IKKs activity and an un-controlled signalling process. Our data suggest that active GSK-3β keeps NEMO phosphorylated and, this way, allows ordered NF-κB activation and recovery of its signalling components.

Particularly the serine residues 17 and 31 of NEMO, identified as targets of GSK-3β in this study, have previously been identified as general phosphorylation sites by phosphopeptide mapping experiments^16^. However, the protein kinases that phosphorylate these sites and modulate NEMO’s function had not been determined. Most GSK-3 substrates require primed phosphorylation^30^. The priming phosphorylation on a Ser/Thr residue by another kinases increases the rate of phosphorylation of another Ser/Thr residue by GSK-3 located 4 amino acids upstream of the primed phosphorylation site. However, a previous report has shown that primed phosphorylation is dispensable when a serine or threonine amino acid residue is flanked by proline residues, as is the case for Ser17/Ser31/Ser43, and when GSK-3 forms complexes with its substrate^7^, as is the case for NEMO, as well. For example, c-Myc Thr58 has been shown to be an *in vivo* phosphorylation site of GSK-3. It is flanked by Pro57 and, interestingly, mutation of Pro57 abolished phosphorylation of Thr58 within the c-Myc protein^31^,^32^. In our study, Ser17 and Ser31 were confirmed to be essential amino acids for GSK-3β-dependent phosphorylation of NEMO, as mutations of these residues to non-phosphorylatable alanines led to the loss of NEMO phosphorylation by the enzyme.

Ser8 and Ser43 phosphorylation of NEMO, documented in this study, has not been reported previously. Although Ser43 flanked by Pro42 may represent a motif for direct phosphorylation by GSK-3, an assumption which was supported by the reduced phosphorylation signal obtained in cells treated with the GSK-3 inhibitor SB216723 or using a GSK-3β kinase inactive mutant, mutation of NEMO at Ser43 had no effect on NF-κB activation by TNFα stimulation. Mutation of Ser8 of NEMO resulted in an even increased phosphorylation at other positions. The significance of either finding was not focus of this study but warrants further investigation as does the search for the phosphatase that dephosphorylates NEMO. Indeed, we performed several mass spectrometric analyses with wild-type and mutant NEMO and have not yet yielded results regarding a phosphatase which can be involved. Nevertheless, ordered activity of NF-κB signaling pathway in addition to phosphorylation is strongly regulated by dephosphorylation and kinase/phosphatase balance. For example, PP2A is a prominent phosphatase in dephosphorylation of NF-κB subunits and has been shown to directly and in cooperation with WIP1 phosphatase dephosphorylate p65^33^,^34^. In addition, PP2A has been shown to be involved in the regulation of the Ser68 phosphorylation of NEMO^11^.

Given the ubiquitous expression throughout various cell types and its role in multiple cellular functions^35^, GSK-3β has been implicated in various diseases such as Alzheimer’s disease, cardiovascular diseases, tumour growth, and many inflammatory diseases^36^,^37^,^38^,^39^. The kinase promotes the production of inflammatory molecules and cell migration, which makes GSK3β a powerful regulator of inflammation. The mechanism by which GSK3β modulates inflammation has been shown to be mostly dependent on NF-κB^39^. For example, inhibition of GSK3β results in impaired NF-κB activation in hepatocytes^40^ and mice lacking GSK-3*β* die during development off multifocal haemorrhagic degeneration of the liver due to defective NF-κB signalling with subsequent unopposed TNFα triggered apoptosis^8^. Likewise, NEMO-deficient mice are characterized by early fatality around embryonic day 12 in males^6^. Death is due to massive liver apoptosis, as well, a phenotype that seems a common theme in knock out models involving critical components of NF-κB signalling, such as RelA or IKKβ^1^. We now provide the direct biochemical link between GSK-3β and NEMO towards NF-κB signalling.

Complex formation with its substrates is a characteristic feature of GSK-3, which warrants that only a specific pool of GSK-3, related to a specific signaling pathway, is active or inactive upon a specific stimulus^41^,^42^. To date, we cannot exclude that other proteins are involved in this complex formation, as already shown for other subunits of NF-κB signaling. For example, interaction of IKKs with p65 is modulated by Rap1 and is crucial for the ability of IKKs to phosphorylate, the p65^43^. Furthermore, GSK-3β has been shown to result in either stabilization or destabilization of its different protein substrates. For example, constitutive GSK-3β phosphorylation of β-catenin leads to its degradation through the ubiquitin/proteasome pathway^44^. Vice versa, constitutive GSK-3β phosphorylation of a specific target protein has been shown to be necessary for its stabilization, too^45^,^46^,^47^,^48^. This is in accordance with our study demonstrating dependency of NEMO protein stability on continuous phosphorylation by GSK-3β. However, K48-linked ubiquitination seemed not involved in degradation of NEMO when not phosphorylated by GSK-3β. Instead, mutation of GSK-3β phosphorylation sites or inhibition of GSK-3 led to an increase of K63-linked NEMO polyubiquitination, even in resting conditions and without TNFα stimulation. K63-linked polyubiquitination does not primarily induce proteasomal degradation but is a crucial prerequisite of IKK complex activation^49^,^50^. It is conceivable that increased K63-linked polyubiquitination of NEMO promotes its recruitment to the IKKs complex with subsequent IκBα degradation and NF-κB activation. Our data imply that through this mechanism the GSK-3β-mediated phosphorylation status of NEMO determines NF-κB activational status. Our finding is in accordance with a recent study demonstrating that inhibition of GSK-3β in quiescent cells resulted in activation of NF-κB through degradation of IκBα^51^. It was postulated that GSK-3β phosphorylates IκBα directly and, this way, induces its degradation^52^. A direct influence of GSK-3β on IκBα as part of the findings downstream of NEMO in our study is conceivable, as well.

Besides modulation of NF-κB activity, K63-linked polyubiquitination of NEMO triggers its p47-mediated lysosomal degradation, as well, thereby putting a negative feedback onto further IKK activation^27^. If mutated and not phosphorylated by GSK-3β, K63-linked polyubiquitination and binding of NEMO to p47 was found further increased in our study implying that through this mechanism GSK-3β not only influences NF-κB activity but NEMO stability, as well.

In summary, our data suggest that the function and stability of NEMO is critically modulated by phosphorylation through GSK-3β at specific sites and emphasise NEMO’s tight regulation through post-translational modifications. The study has important implications for the development of novel therapeutic approaches towards inflammatory diseases that are characterised by excess and/or prolonged NF-κB activation.

## Materials and Methods

### Reagents and antibodies

The following antibodies were used: anti-GSK-3β (#9832), anti-K48-linked ubiquitin (#8081), NF-κB Pathway Sampler Kit (#9936), anti-TNF-R1 (#C25C1) and anti-RIP (#D94C12) (New England BioLabs, Frankfurt/Main, Germany), anti-Ubiquitin from DAKO (Hamburg, Germany), anti-pSer-31-NEMO, anti-TRAF6, anti-RIP1 and anti-β-actin (Abcam, Cambridge, UK); anti-p47 (Abnova, Heidelberg, Germany); phospho-specific anti-pSer-8-NEMO and anti-pSer-17-NEMO (Eurogentec, Köln, Germany); anti-FLAG M2 mouse (Sigma-Aldrich, Taufkirchen, Germany); anti-pSer-43-NEMO (Abgent, Heidelberg, Germany); anti-Tubulin and non-immune IgGs (Invitrogen, Darmstadt, Germany). Recombinant human TNFα was from Miltenyi (Bergisch Gladbach, Germany), GSK-3 inhibitors SB216730 and AR-A014418 from Calbiochem (La Jolla, CA, USA).

### Cell culture

All culture media and supplements were purchased from PAA Laboratories (Coelbe, Germany). HEK293, MCF-7, NIH3T3 and peripheral blood mononuclear cells cells were grown as described^48^,^53^. Peripheral blood mononuclear cells (PBMNCs) were isolated as described^53^. Adherent cells were further cultivated for 14 days to obtain monocyte-derived macrophages. All procedures involving human materials have been approved by the local Ethics Committee of University Magdeburg complying with the principles of the Declaration of Helsinki. Informed consents were obtained from all patients.

### Luciferase assay

HEK293 cells stably transfected with 5xNF-κB-RE (Promega, Mannheim, Germany) were washed with phosphate-buffered saline (Mg^2+^- and Ca^2+^-free) and lysed in 150 μl/well luciferase cell culture lysis reagent (Promega, Mannheim, Germany). Luciferase assays were performed using the luciferase assay system from Promega, according to the manufacturer’s instructions, and quantified with a luminometer (LB9506, Berthold, Bad Wildbad, Germany).

### Immunoprecipitation

Immunoprecipitation was performed as described previously^48^. In addition, for the isolation of GFP-tagged proteins we used the GFP Isolation Kit (#130-091-125), according to the manufacturer’s instructions (Miltenyi, Bergisch Gladbach, Germany).

### Transfection

Transfection of HEK293 cells with siRNA was performed using Viromer (Lipocalyx, Halle, Germany) according to the manufacturer’s instructions. siRNA oligonucleotides were purchased from Sigma Aldrich (Sigma-Aldrich, Taufkirchen, Germany). The following siRNA sequences were used: siGSK-3β: EHU0794515, SIHK0873, SIHK0872, siGSK-3α: EHU040791, SIHK0869. The plasmids for GSK-3β have been described previously^48^. The full-length cDNA of human NEMO was cloned into pTagGFP-N or pTagRFP-N (Evrogen). Site-directed mutagenesis of the NEMO (serine to alanine or aspartic acid) was performed using the QuikChange site-directed mutagenesis kit from Stratagene (La Jolla, CA, USA). Mutations were verified by DNA sequence analysis. Ubiquitin wild-type (Addgene plasmid 17606; Cambridge, UK) and mutated ubiquitin, Ub-K48 and Ub-K63 (Addgene plasmids 17605, 17608), were used and have been described elsewhere^54^.

### *In vitro* phosphorylation assay

Recombinant human IKKγ (Abnova, Heidelberg, Germany) or purified full-length GST-IKKγ-fusion protein (wild-type and mutants) were incubated with GSK-3β (New England BioLabs) or IKKβ at 30 °C in a total volume of 30 μl of GSK-3β kinase assay buffer containing 10 μCi of [γ-32P]ATP (5000 Ci/mmol). Phosphoprotein products were detected by PAGE (10% gel), Coomassie Blue staining, and autoradiography.

### Real-Time RT PCR

RNA isolation and Real-Time PCR were performed as described previously^55^, using the following primers: IL8: forward, 5′-CTTCAAAAACTTCTCCACAAC-3′, and reverse, 5′-GGACAAGAGCCAGGAAGAAACC-3′; IL1β: forward, 5′-AGAATCTGTACCTGTCCTG-3′, and reverse, 5′-CTTGAGAGGTGCTGATGT-3′; HPRT1: forward, 5′-TTGCGACCTTGACCATCTTTG-3′, and reverse, CTTTGCTGACCTGCTGGATTAC.

### Widefield FLIM Setup

The Widefield FLIM set-ups used for our FRET experiments have been described previously^56^.

### Cell culture staining for STED-microscopy

HEK293 cells were fixed using 4% paraformaldehyde for 10 min at RT, followed by 3 times washing with PBS. Incubation with the primary antibody was performed overnight at 4 °C. Primary antibody was removed; cells were washed 5 times with PBS and then incubated with fluorescence-labeled secondary antibody in blocking buffer. The following secondary antibodies were used: Anti-rabbit ATTO 647N, dilution 1:200, and anti-mouse Chromeo 494, dilution 1:50 (Active Motif, La Hulpe, Belgium). Incubation was carried out at RT for one hour followed by another 5 times of washing. Finally, cells were embedded in Mowiol, pH 8.4.

### Image acquisition of stained cell cultures by STED-microscopy

For confocal and STED-imaging of Chromeo 494 and Atto 647N dyes with emission maxima around 600 nm (channel 1) and 680 nm (channel 2), respectively, we used a 2-channel TiSphr.-pulsed STED microscope (Leica Microsystems, Germany) equipped with a 100 x Oil Plan Apo NA 1.4 STED objective. Confocal as well as STED images were recorded by scanning the focused beam with a galvo-mirror at 1000 Hz with 64 line average with a pixel size of 25 nm across the specimen. In STED mode, a pixel size of 25 nm was achieved with the 100 x Oil NA 1.4 objective at an image format of 1024 pixel using a zoom factor of 6.

Typically, an image series consisted of 4 channels. Channel 1: Atto 647N (LSM mode), channel 2: chromeo 494 (LSM mode), channel 3: Atto 647N (STED mode), channel 4: Chromeo 494 (STED mode).

#### Image Processing

To improve image quality raw data of confocal and STED images were deconvolved using Autoquant deconvolution software (Media Cybernetics Inc., Bethesda, USA) with a theoretical PSF. Subsequently, images were processed using ImageJ (National Institutes of Health, USA) for merging channels and conversion into 8 Bit RGB images. Contrast and brightness levels of individual channels were finally adapted by Photoshop CS 5 (Adobe. System Inc., San Jose, USA).

### Statistical Analysis

Data are given as mean ± SEM. Statistical analysis was performed by ANOVA. Post-test multiple comparison was performed by the Bonferroni method. All experiments were independently repeated at least three times.

## Additional Information

**How to cite this article**: Medunjanin, S. *et al*. GSK-3β controls NF-kappaB activity via IKKγ/NEMO. *Sci. Rep.* **6**, 38553; doi: 10.1038/srep38553 (2016).

**Publisher’s note:** Springer Nature remains neutral with regard to jurisdictional claims in published maps and institutional affiliations.

## Supplementary Material

Supplementary Figures
