# Supplementary material for: GSK-3β controls NF-kappaB activity via IKKγ/NEMO
Source: Sci Rep. 2016 Dec 8;6:38553. doi: 10.1038/srep38553 (PMC5144080; doi:10.1038/srep38553)
Supplement: Supplementary Figures [file srep38553-s1.pdf]

GSK-3β controls NF-kappaB activity via IKKγ/NEMO

Senad Medunjanin[1], Lisa Schleithoff[1], Christian Fiegehenn[1], Soenke Weinert[1], Werner Zuschratter[2], Ruediger C. Braun-Dullaeus[1]

[1]Internal Medicine/ Cardiology and Angiology, Magdeburg University, Magdeburg, Germany,
[2]Leibniz Institute for Neurobiology, Magdeburg, Germany.

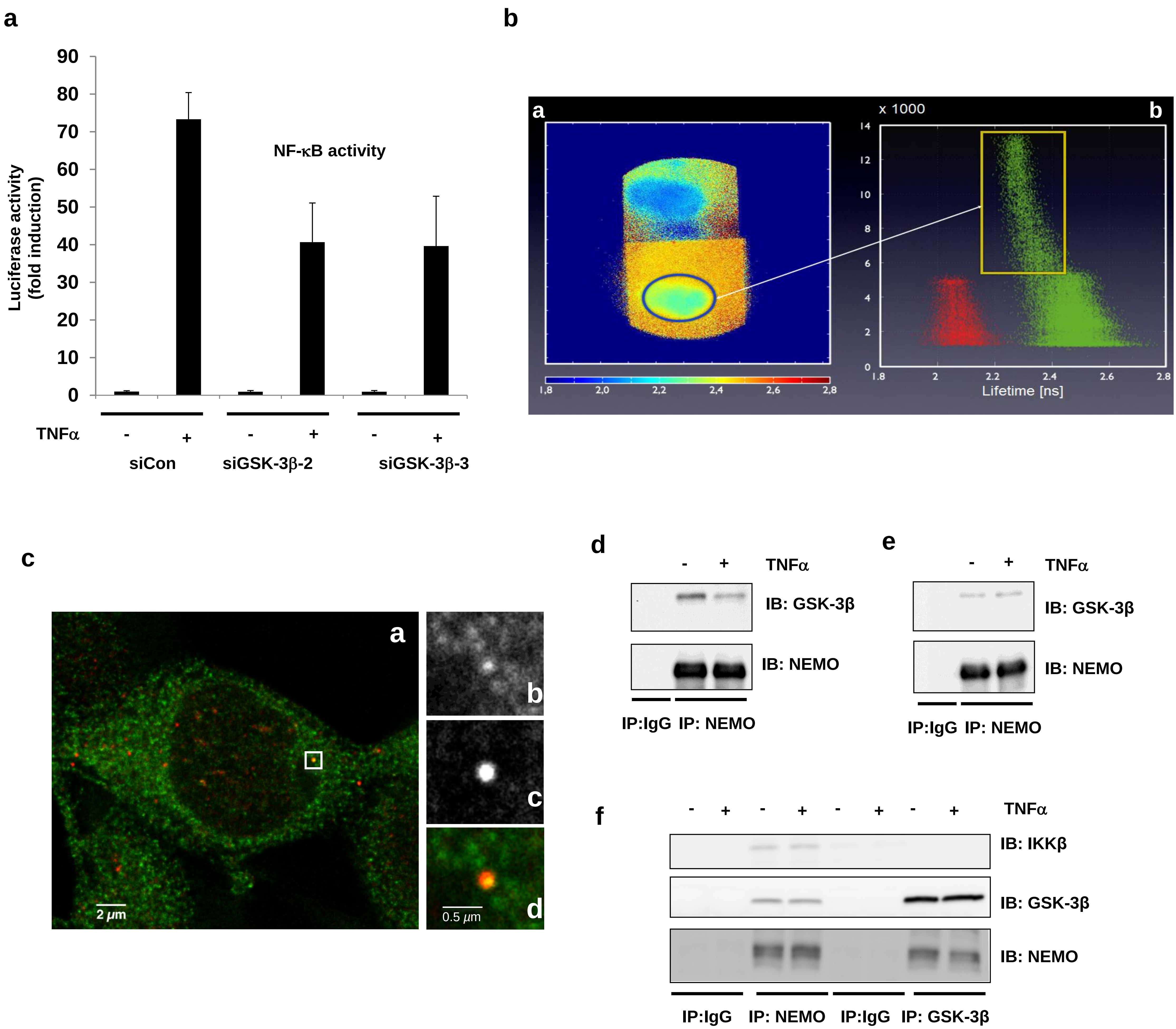

Supplementary information, Figure S1.

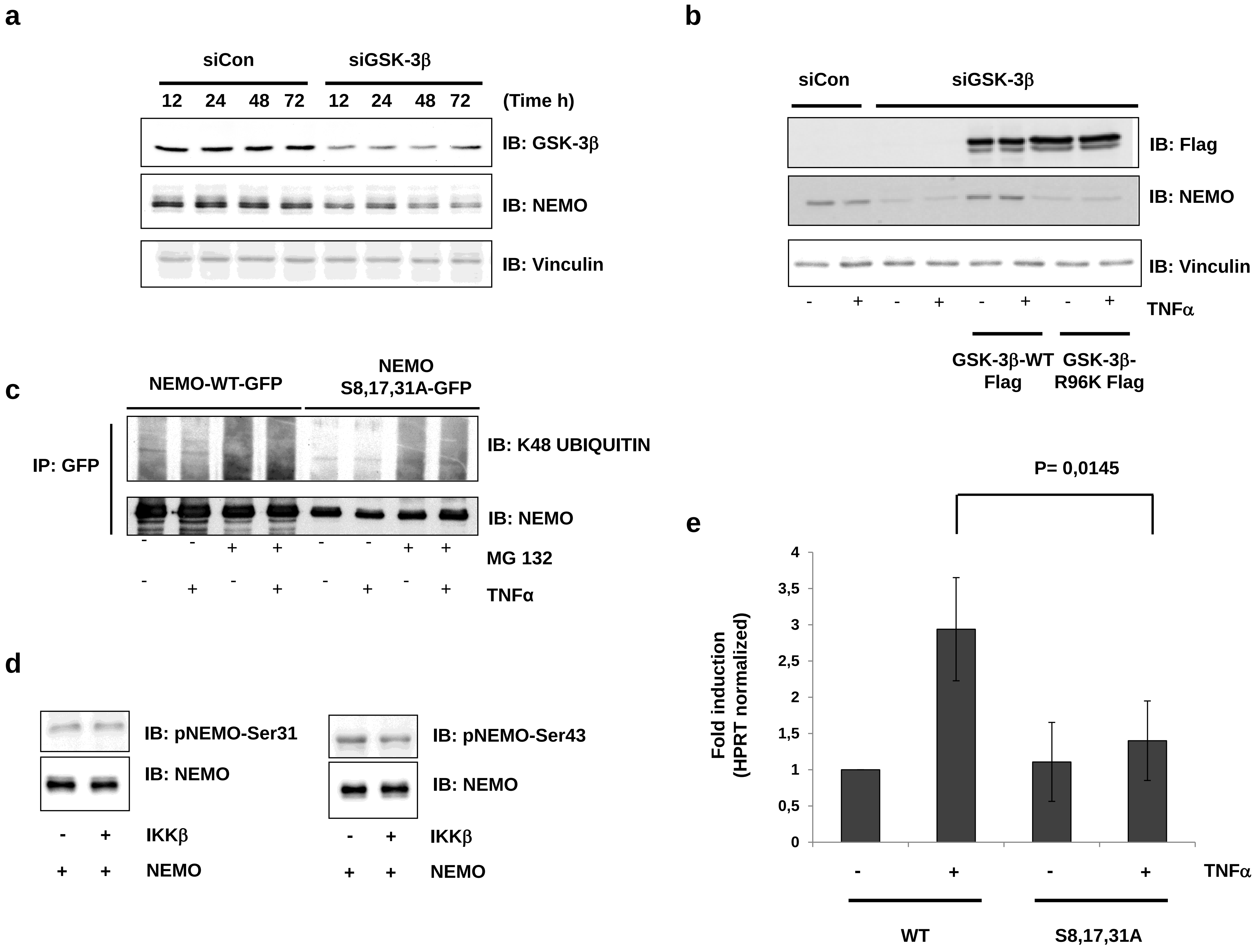

Supplementary information, Figure S2.

Supplementary Figure Legends

Supplementary information, Figure S1. (**a**) FLIM image separated by donor and acceptor lifetimes (a). Note the reduction in donor-lifetime within the region of interest compared to the surrounding cytoplasm. (b) Lifetime plots of donor and acceptor channels shown in (a). (**b**) Distribution of endogenous NEMO (red) and GSK-3β (green) visualized by immunostaining of HEK293 cells and subsequent STED imaging. Boxed area in (a) is magnified in (b) GSK-3β, (c) NEMO, (d) Overlay. Whereas NEMO-immunoreactivity was found in "speckles" within the cytoplasm, GSK-3β exhibited a disperse distribution throughout the cytoplasm, where it was associated with NEMO. (**c**) CoIP of NEMO and GSK-3β from lysates of MCF-7 cells after stimulation with TNFα (10 ng/ml) for 30 min. (**d**) CoIP of NEMO and GSK-3β from lysates of macrophages after stimulation with TNFα (10 ng/ml) for 30 min. (**e**) CoIP of NEMO and GSK-3β from lysates of NIH3T3 cells after stimulation with TNFα (10 ng/ml) for 30 min.

Supplementary information, Figure S2. (**a**) HEK293 cells were transfected either with GL3 control siRNA or with siRNA targeting GSK-3β. After incubation for different time periods cell lysates were immunoblotted with the antibodies indicated. (**b**) HEK293 cells were transfected either with GL3 control siRNA or with siRNA targeting GSK-3β. After 24 incubation, cells were additionally transfected with wild-type GSK-3β or mutant GSK-3β-R96K and cell lysates were assayed for the expression of the proteins as indicated. (**c**) HEK293 cells were transfected with wild type-NEMO or with the S8A, S17A, S31A mutant of NEMO. After 6 h pretreatment with MG132 (5 μm), cells were treated with TNFα (10ng/ml) for 1 h. The lysates were immunoprecipitated (IP) with anti-GFP, followed by immunoblotting (IB) with anti-K48 ubiquitin. (**d**) *In vitro* kinase assay using recombinant human NEMO (1 μg) as substrate for IKKβ (0.01 μg). The same amounts of proteins are immunoblotted with the antibodies as indicated. (**e**) HEK293 cells were transfected either with wild-type NEMO or with mutated NEMO. The cells were treated—or not—with TNFα (10 ng/ml) for 24 h, and analyzed for the expression of NF-κB-target gene, IL1β by quantitative RT–PCR
